# Detection of Barely Visible Impact Damage in Polymeric Laminated Composites Using a Biomimetic Tactile Whisker

**DOI:** 10.3390/polym13203587

**Published:** 2021-10-18

**Authors:** Sakineh Fotouhi, Saber Khayatzadeh, Wei Xia Pui, Mahdi Damghani, Mahdi Bodaghi, Mohamad Fotouhi

**Affiliations:** 1 School of Engineering, University of Glasgow, Glasgow G12 8QQ, UK; Sakineh.Fotouhi@glasgow.ac.uk; 2 Department of Design and Mathematics, University of the West of England, Bristol BS16 1QY, UK; saber.khayatzadeh@uwe.ac.uk (S.K.); Mahdi.damghani@uwe.ac.uk (M.D.); 3 Department of Aerospace Engineering, Bristol Composites Institute (ACCIS), University of Bristol, Bristol BS8 1TR, UK; weixiapui@gmail.com; 4 Department of Engineering, School of Science and Technology, Nottingham Trent University, Nottingham NG11 8NS, UK; mahdi.bodaghi@ntu.ac.uk

**Keywords:** composite materials, damage detection, low velocity impact, whisker, ultrasonic

## Abstract

This is a novel investigation on the possibility of detecting barely visible impact damage (BVID) in composite materials by whisking across the surface via tactile whisker sensors that resemble rats’ whiskers. A series of drop tower low-velocity impact tests were performed on quasi-isotropic composite plates. The plates were made from unidirectional T800 carbon/MTM49-3 epoxy prepregs with the stacking sequence of [45/0/90/−45]_4S_. Investigating the specimens’ surface by the naked eye does not reveal any significant damage, rather than a small dent on the surface, with no tangible difference in the different impact energy levels. Ultrasonic C-scan observations showed the existence of BVID in all the impact energy levels, with an increasing trend in the damage size by increasing the impact energy level. The collected data from whisker sensors were analyzed using the support vector machine classifier, based on their vibrational properties, to identify the impacted region and classify the impact severity. It was observed that after training for 13 whisker contacts, the BVID severity can be classified with an accuracy of 100%. This is offering a new BVID detection technique, with a high potential for automation and high reliability that can be used as an alternative or combined with available inspection systems.

## 1. Introduction

The demand for composite laminated materials has increased significantly over the last decade. In the past, the application of such materials was limited to the aerospace industry. However, in recent years, composites have been widely used in other sectors amongst which are sport, rail, automotive, marine, defence, energy, and construction industries. The key advantage of such materials is their high strength to weight ratio making them ideal for use as lightweight and high load bearing structural components. However, poor out-of-plane properties, low damage tolerance and low delamination resistance have limited their application as primary loading components [1].

Impact damage may take place because of either high velocity or low velocity foreign objects. Impact damage leads to delamination (separation of plies), matrix cracking and fibre breakage [2]. This degrades both strength and stiffness reducing load bearing capacity of the composite laminate structure [3,4,5]. Therefore, if the introduction of the damage is undetected, premature and catastrophic structural failures could be expected.

**Figure 1:**
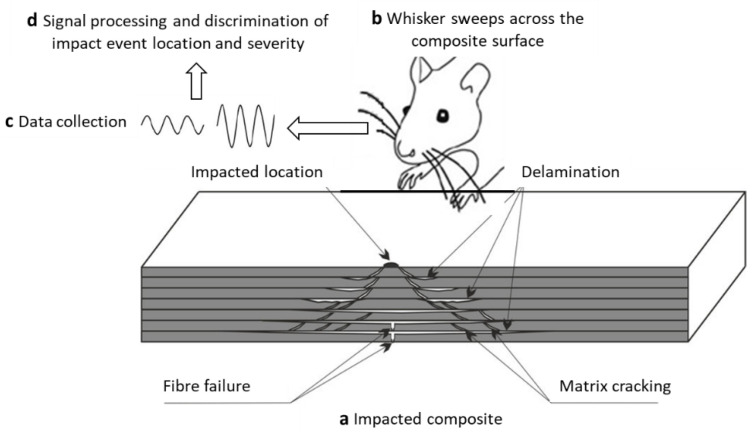
Overview of the project, and a schematic of a typical damage mechanisms in BVID.

The damage caused by high velocity impact, i.e., ballistic impact, is highly localised at the point of impact and encompasses all of the previously mentioned failure modes, i.e., delamination, matrix cracking and fibre breakage, and is visible to the naked eye. Therefore, it can be detected by routine visual inspections. On the other hand, Low Velocity Impact (LVI) leads to Barely Visible Impact Damage (BVID). The source of impacting foreign objects depends on where the composite laminate is used. For example, in the case of an aircraft, such damage is often caused by debris or bird strike during landing or take-off and hailstorm when in flight. Tools dropped accidentally during assembly or maintenance can also contribute towards the formation of BVID [6]. BVID is dominated by matrix properties, where matrix cracking takes place at inter-lamina locations followed by delamination [7], see Figure 1. Such damage cannot be detected in routine visual inspections and can pose a challenge to structural integrity. Therefore, Non-destructive Testing and Evaluation (NDT&E) are needed for damage determination and quantification leading to a proper repair or ‘use-as-is’ decision.

NDT&E strategies can be approximately classified as [2,8,9]:

- Visual inspection;
- Sonic and ultrasonic (guide wave, laser ultrasonics, tap test, acoustic emission, etc.);
- Optical (digital image correlation, shearography etc.);
- Optical thermography (pulsed phase and line scanning thermography, etc.);
- Non-optical thermography (eddy current and microwave thermography, etc.);
- Electromagnetic (eddy current, inductive, capacitive, microwave, terahertz, etc.);
- Radiographic (X-rays, gamma-rays).

All these NDT&E methods have their advantages and disadvantages. Sonic and ultrasonic techniques are the primary NDT&E methods utilised by the industry as quality assurance checks for the production of composite parts and structures. Ultrasonic test, particularly by means of phased arrays, is used for detecting discontinuities such as delamination since the damage is an effective reflector of ultrasonic waves. However, such inspection techniques require labour intensive and time consuming inspection of parts [10]. This proves even more difficult if inspection of inaccessible or large structures such as wind turbine blades, aircraft fuselage or wing is intended. Furthermore, ultrasound techniques have limitations when testing thin layered structures arising from the necessity to use high frequencies (in the range of tens of MHz), as there is a high attenuation in composite at high frequencies. A large number of data and noise signals are also considered as big challenges while using acoustic emission inspection [11].

Optical based techniques such as shearography measure the distance between adjacent points in a specimen under loading. The measured displacements decide whether there are defects in the material. The key advantage of optical methods is that they are capable of inspecting a large area in a relatively short time. However, the parameters of multilayer 3D materials cannot be observed by optical systems due to the limitation of surface/subsurface inspection. For example, such techniques are not usually suitable for delamination detection as there is not enough deformation to detect on the surface [12].

Optical thermography techniques work on the basis of heat induction from the surface through the depth of material. Excitation of material could be as a result of thermal or optical sources such as lamps or lasers. The presence of a defect on the surface or subsurface disrupts locally heat induction between the layers which generates a thermal perturbation at the surface of the composite. This perturbation leads to different amplitudes and phase shifts of the thermal wave in the defected zone of the specimen with respect to the intact one. Therefore, the defect can be detected by the temperature difference contrast or the phase contrast [13,14,15].

Since Carbon Fibre Reinforced Polymers (CFRP) are anisotropic conductive material, electromagnetic techniques such as eddy currents and microwave thermography could provide invaluable information regarding the internal structure of laminated composites [16]. In such techniques, the structure is excited by an external magnetic field, usually by means of sinusoidal alternating current through a coil. The electromagnetic field then penetrates into the conductive material. The change in impedance of coil represents the discontinuity in material conductivity as a result of the existence of dents, cracks, and delamination. However, the extent of penetration of electromagnetic waves depends on the inspection frequency of the waves which is limited to 3 kHz to 300 GHz. This limits the thickness of the laminate to be inspected [17].

Radiographic testing techniques [18] are based on transmitting ionizing radiation through the material and measure its attenuation. They allow the detection of internal flaws and defects such as cracks, corrosion, inclusions, and thickness variations. However, they not only pose radiation hazard but also have limited capacity in detecting delamination and planar cracks. In such techniques, different projections at different angles are measured and a full 3D reconstruction is performed. As such, to detect delamination, a resolution comparable to its thickness is required, which for large components is a demanding requirement.

All the aforementioned techniques, considering their pros and cons, provide useful information regarding the internal flaws of laminated composite structures after BVID is taken place. However, their application becomes challenging and labour intensive if the point of impact is not known on large structures such as wings and fuselage of aircraft or wind turbine blades. In addition, most of these NDT&E techniques require high levels of operator experience, and are usually expensive, time-consuming, and sophisticated. In some cases, further inconvenience may rise as the component has to also be out of service for the inspection. As a result, in this paper we have tried to provide a new bio-inspired inspection solution for composite structures with a potential for automation and to lower the cost and reliability of inspection. Figure 1 shows the overview of this project, where application of biomimetic whiskers is investigated for BVID detection and distinguishing between different levels of impact energy. This method provides a good potential for automated inspection of composite structures, especially for large-scale structures in different sectors such as aerospace and wind energy. This can reduce health and safety risks and surpass human accuracy, if combined with the recent advances in sensors [19,20], automation [21], data analytics [22], and artificial intelligence technologies [23,24,25].

This is the first time that the whisker technology is used in BVID inspection in composites, hence there is no published work to review. Therefore, we have focused mainly on the biological inspiration and the development of the whisker sensor which was used in this work. Rats can accurately discriminate textures based on one to three touches per whisker [26]. In rats, whiskers are curved and taper from a diameter of less than 1 mm at the base to a narrow tip. When a whisker’s tip or shaft contacts a texture, its movement changes; whisker motion signals are brought together in each of the primary afferent neurons of the brainstem trigeminal nerve where they are relayed to the rest of the brain. One of the most interesting properties of the rat whisker system is that the macrovibriasse are not passive sensors waiting to be deflected by an object but rather the whiskers are actively swept back and forth at high speeds in a behaviour known as whisking where the forward movement of each whisker is determined by its own muscle [27]. These interesting properties of rat whiskers drove the research towards creating robotic vibrissal systems that can mimic the properties of actual rat whiskers. In order to do that biomimetic whiskers mimic the design of rat whiskers by having an approximately round cross-section which decrease in width along the length of the whisker till the tip and have no sensors along the length of the whisker, but have the sensing done in the follicle at the base of the whisker [28]. To ensure the transmission of the tactile information to the follcile it was found that the tapering of the whiskers was very important [29]. Kim and Möller [30] attempted to use a biomimetic whisker for shape recognition. The work completed in the paper explored the concept that the deflection angle or velocity of a whisker provides the localization information which is the basis of shape recognition. Shape recognition was successfully achieved using several types of objects consisting of circular or square discs by measuring the deflection size and angle of the whisker during active whisking. Since a square object has an edged surface and a round shaped object has a curved surface, comparing the two sets of deflection data was shown to be able to provide appropriate shape information. Evans et al. [27] took a different approach to the manufacturing of the whisker sensor by 3D printing the whisker using flexible ABS plastic. Further, 3D printing was used as it would allow for the whisker to be tapered unlike whiskers simulated using a rod of fixed diameter. In this paper whisker sensors that were developed by Sullivan et al. [31] using 3D printing technique was used for the investigation, which is a good resemblance of rat’s whisker system. This paper for first time reports a novel application of the tactile whisker sensors on the possibility of detecting BVID in laminated composite materials.

## 2. Experimental Methods

### 2.1. Manufacturing and Impact Test

Unidirectional T800 carbon/MTM49-3 epoxy prepreg supplied by Solvay (Derby, UK) was used to fabricate 330 × 330 mm^2^ square plate that then was cut to the test sample size, i.e., a rectangular plate with nominal in-plane dimension of 135 × 93.5 mm^2^ in-plane and 4.65 mm thickness. Properties of the prepreg are summarized in Table 1. The laminate was laid up in a quasi-isotropic stacking sequence, [45/0/90/−45]_4S_, where 0 is the direction of unidirectional fibre orientation parallel to the long side of the plate.

**Figure 2:**
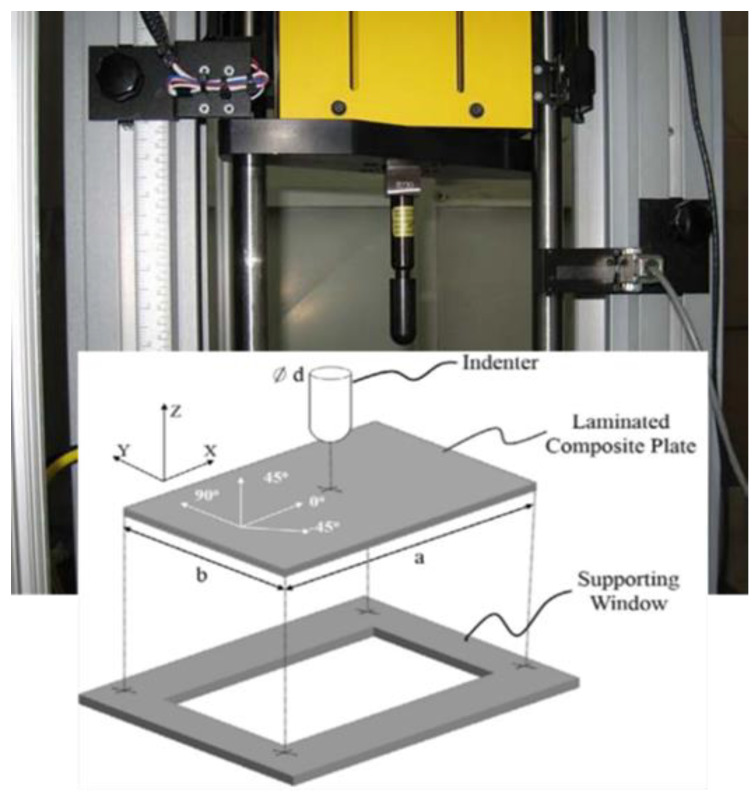
Experimental set up for the LVI tests.

LVI tests were carried out using an Instron Dynatup 9250 HV drop-weight impact tower, manufactured by Instron based in Norwood, MA, USA, and according to the ASTM D7136 standard [32]. The test samples were simply supported on a 125 × 75 mm window with four rubber-tipped clamps, as illustrated in Figure 2. The impact energies used were 8 J, 12 J, 36 J, and 64 J, respectively. The impact load and deflection were measured by a single accelerometer inside the tup, and the measured data were processed by a 4 kHz filter of the console software to reduce the noise and oscillations. Ultrasonic C-scan was used to understand the damage mechanisms occurring during the impact events.

### 2.2. Set-Up of the Whisker Experiment

**Figure 3:**
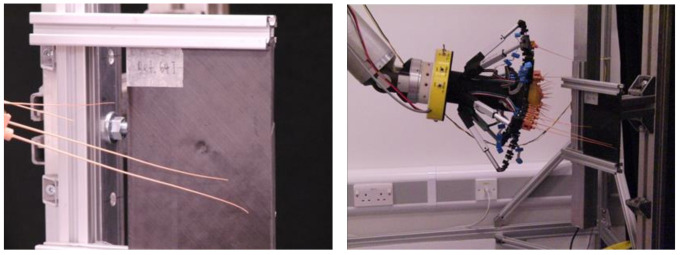
Whisker scanning set-up.

An array of whisker sensors was positioned using a robot manipulator arm (Barrett WAM, Newton, MA, USA), 7 degrees of freedom) and held so that two whiskers could impinge on a single plate as shown in Figure 3.

**Figure 4:**
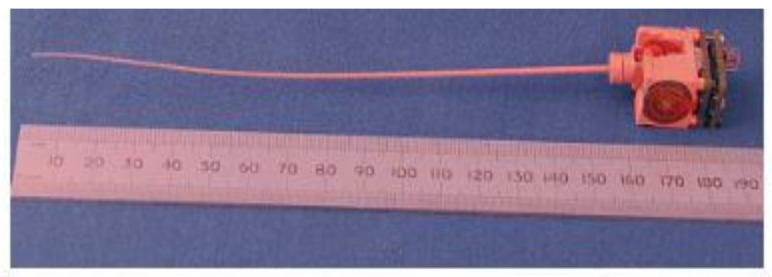
The largest utilized whisker sensor.

Each individual whisker module comprises a small electric motor and a thin tapered plastic whisker: the thicker end of which is connected to the output shaft of the motor. The motor is used to drive the whisker in an oscillatory manner which is inspired by the motion of rat whiskers. In common with rat whiskers, the whisker shaft itself is inert and the sensory transducer is at the driven end of the whisker. A Hall-effect sensor is used to detect rotary deflection of the base of the whisker and the distal tip of the whisker is used to probe the surface under investigation. The base of the whisker widens to form a ball which is located within a spherical socket housing and is retained in place by filling the socket with a soft polymer which also serves as a restoring spring and a vibration damper. The socket, which also houses the Hall-effect sensor, is rigidly attached to the motor drive shaft. The whiskers were 3D-printed using EnvisionTEC RC31 composite material (BRL, Bristol, UK), provided by EnvisionTEC in the UK, and digital light processing technology to achieve a smoothly tapered whisker with fine tip size coupled with sufficient strength and toughness to withstand repeated impact against surfaces (see Figure 4). The largest dimensions of the whiskers used were 155 mm length, 1.5 mm base diameter, and 0.3 mm tip diameter.

The whiskers were actively driven (using an integrated miniature brushless geared DC motor) against the front (impacted) face of the sample plates. As the impact event location was known to be in the centre of the plates, the scanning was taken from the centre, and areas far from the centre where no damage was expected. The corresponding protraction angle (θ) with respect to the axis of the robot end-effector was measured to 14-bit resolution using a second Hall-effect sensor. Real time drive signal generation and closed loop Proportional-Derivative (PD) control is provided by the integrated 16-bit micro-controller (dsPIC33f, (BRL, Bristol, UK), allowing for accurate control at whisking frequencies between 1 Hz and 8 Hz. Orthogonal axes deflections of the whisker shaft base are measured with 14-bit resolution using a Hall-effect sensor which detects the direction of the magnetic field generated by a small permanent magnet attached to the base of the whisker. All three measured variables (θ, x, y) were transferred, at a 2 kHz sampling rate, to a host Personal Computer (PC) via a high-speed USB2 interface. In the classification experiments reported here, only the x displacements were used.

In each test sequence, the material specimen was impacted 20 times by the whiskers at a frequency of 2 Hz to generate training data for the machine learning algorithm. In each sequence, the first and last whisks were discarded as there were observable differences in the data at the start and end of each run, probably due to the control system. Data were collected at 2 kHz for each “whisk”—a movement of the whisker through an angle of about 70 degrees and touching the test surface at a point near to the furthest protraction position in the swing. In this way, the contact is very light, and repetitions are constrained to be as similar as possible. In subsequent data processing and classification procedures, only one whisker was used as it was found that successful classification could be performed with a single whisker. The longest whisker in the row (the one on the left of Figure 3) was used in the classification experiments.

### 2.3. Signal Processing

**Figure 5:**
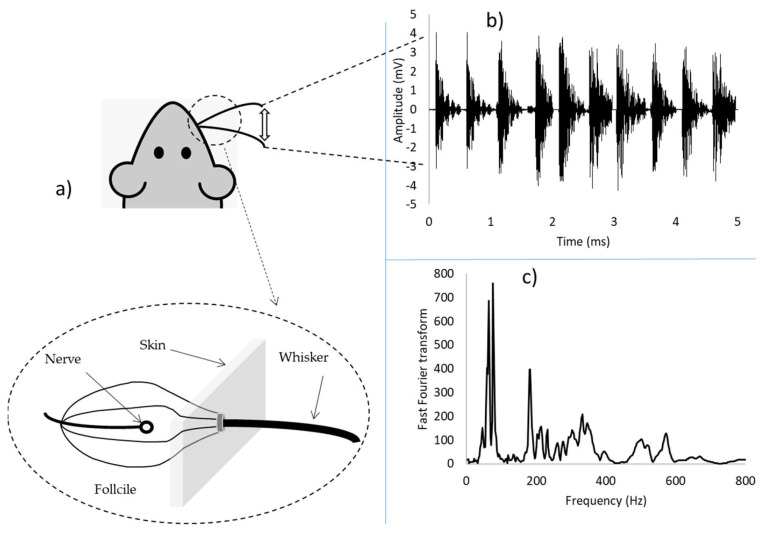
(**a**) Schematic of the whisker sensor, (**b**) Typical transient signals obtained from the whisker sensor, and (**c**) representation of a signal after applying fast Fourier transform analysis.

The approach used aims to take advantage of the non-stationary frequency characteristics of the intermittently contacting whisker signals. In particular, time-frequency analysis, using the Time frequency toolbox [33] to derive feature vectors for classification. Figure 5 shows typical transient signals obtained from the whisker sensor, and a fast Fourier transform analysis on a collected signal.

Equation (1) gives the pseudo-Wigner–Ville distribution of a continuous time series x(t) carried out by adapting the fast Fourier transform algorithm [34]. This distribution was used in this paper for the time-frequency analysis. The distribution gave good results in our classification test, with acceptable computing times.

(1) $$W_{x}(t,f)\;=\;\int w(\tau )x(t+\tau /2)\bar{x\left(t-\tau /2\right)}\;\exp(-2\pi \mathrm{if}\tau )d\tau$$

where w(τ) is a window function, x(t + τ/2)$\bar{x\left(t-\tau /2\right)}$ is the Fourier transform for fixed t as τ varies.

The maximum frequency in this analysis was set at 800 Hz which is less than the Nyquist frequency for the 2 kHz sampling rate. Feature vectors were generated by dividing the time-frequency plane into 80 uniformly sized partitions and computing the mean of the frequency distribution within each partition. These data were normalised before training the classifier.

## 3. Results and Discussion

### 3.1. Mechanical Results

**Figure 6:**
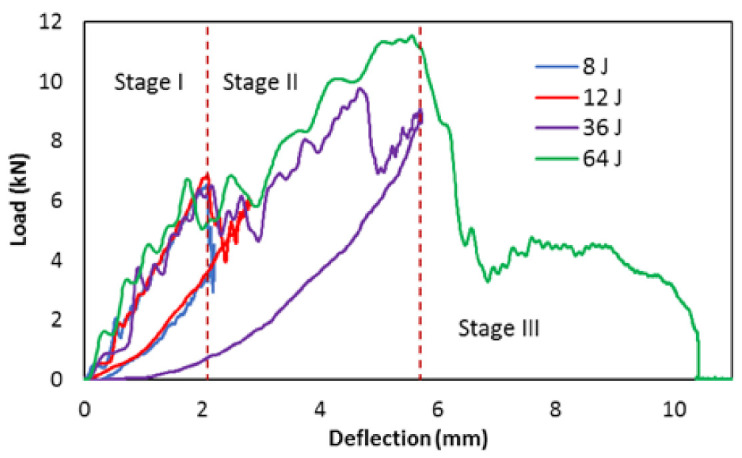
Comparisons of load-deflection plot generated from the drop-weight impact for the investigated laminates at different energy levels.

**Figure 7:**
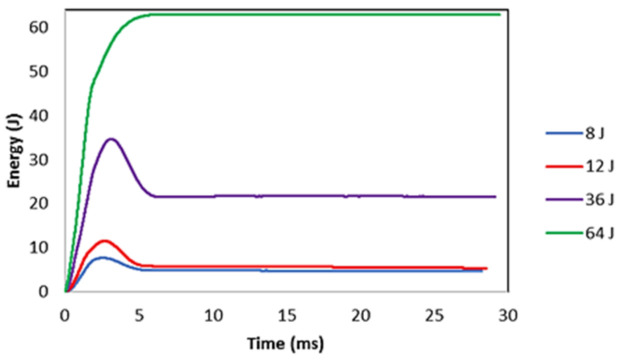
Comparisons of energy-time plot generated from the drop-weight impact for the investigated laminates at different energy levels.

Figure 6 and Figure 7 show comparisons of energy absorption and load-deflection plots obtained during the LVI under increasing impact energies. The data were extracted from Instron Dynatup 9250 HV drop-weight impact tower (Norwood, MA, USA), where a piezoelectric force transducer was used to measure the load, a position transducer and optical encoder were used to measure displacement and velocity. Three stages of the load-deflection can be seen from the load-deflection plots in Figure 6. Stage I is associated with linear behaviour in the early stage of the loading process with an elastic response, and no underlying delamination damage in the laminate. Stage II is related to the initiation and unstable propagation of BVID (mainly delamination) and is distinguishable from the load drops. With the increasing load in Stage II, the number and size of delamination increases. In stage III there is another load-drop due to fibre failure at the back face of the specimen that is under tension. The impact energies were chosen to induce BVID to check the ability of the whisker technology in detecting such damage. The specimens impacted with 8 J, 12 J, and 36 J experience just Stage I and Stage II, due to the lower level of impact energy that causes initiation and propagation of delamination. In these impact energies, the specimens are still integral, and the deflection goes close to zero after the impact. In the 36 J case, there is a second load drop on the load in Stage II that corresponds to a second unstable delamination propagation that is presented in the post-impact C-scan results in the next section. For the specimen impacted with 64 J, all the three stages exist. This specimen experienced delamination initiation, propagation and fibre breakage in the tension side.

The energy absorption in composite laminates is through the damage mechanism. Comparing the absorbed impact energy at different energy levels, the induced damage in higher impact energy levels is clearly reflected by a difference in absorbed energy (see Figure 7). For the 64 J, the highest impact energy absorption and consequently the highest damage level is expected.

### 3.2. Visual Observations and C-Scan Results

**Figure 8:**
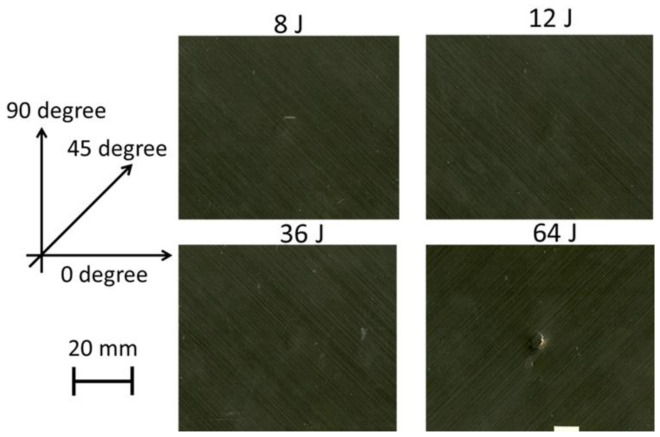
Images of samples that taken from the impacted surface at different impact energy levels using EPSON scanner.

**Figure 9:**
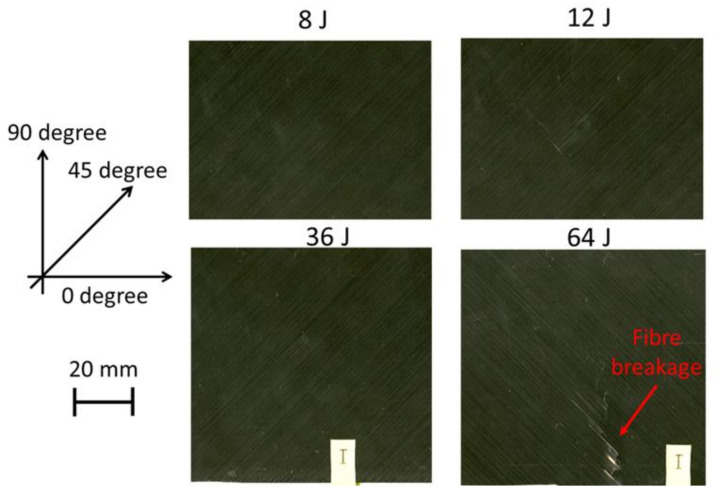
Images of samples that taken from the back face at different impact energy levels using EPSON scanner.

**Figure 10:**
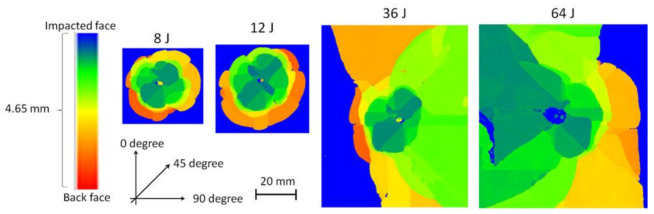
C-scan images of the laminate with various impact energies.

An EPSON scanner, supplied by EPSON in the London, UK, was used to take clear pictures of both the impacted face (Figure 8) and the back face of the samples (Figure 9) after the LVI test at different energy levels. The visual impact damage of the samples is shown on both the front and back surfaces. The impacted side of the samples does not reveal any significant damage, rather than a small dent, with no tangible difference in the different impact energy levels. It is the same for the back face of the laminates, except for the 64 J that an obvious fibre breakage is observable due to the high tensile strains. Comparing the visually observable pictures in Figure 8 and Figure 9 with the C-scan results illustrated in Figure 10, there is a huge difference between the internal damage and the visible damage on the impacted face. The C-scan results confirm the existence of the BVID in all the impact energy levels, with an increasing trend in the damage size by increasing the impact energy level.

### 3.3. Results for the Tactile Whisker

The classification was performed using a Support Vector Machine (SVM) classifier, implemented using LIBSVM and GNU Octave [35], developed by the National Taiwan University. Classifiers based on SVMs have few free parameters requiring tuning, are simple to implement, and are trained through optimization of a convex quadratic cost function, which ensures the uniqueness of the SVM solution [36]. A Radial Basis Function (RBF) kernel was used. The SVM is essentially a binary classifier but in this example, we have four classes corresponding to the four levels of impact energy tested. Hsu and Lin [37] discussed a number of ways of extending SVM classification to perform multi-class classification. Here we have chosen to use the simple “one-against-one” method which is available as an option in libsvm and is generally faster to train than the other options.

The training was a two-stage process: an initial stage used 5-fold cross-validation testing with a grid-search to find optimal values of the two free SVM parameters: C and gamma. The second stage used these optimal values and a probability model to form a predictive model which was used in the validation testing procedure. Training data was obtained by palpating each specimen 18 times giving a total of 72 training data sets, each containing 1000 time points.

**Figure 11:**
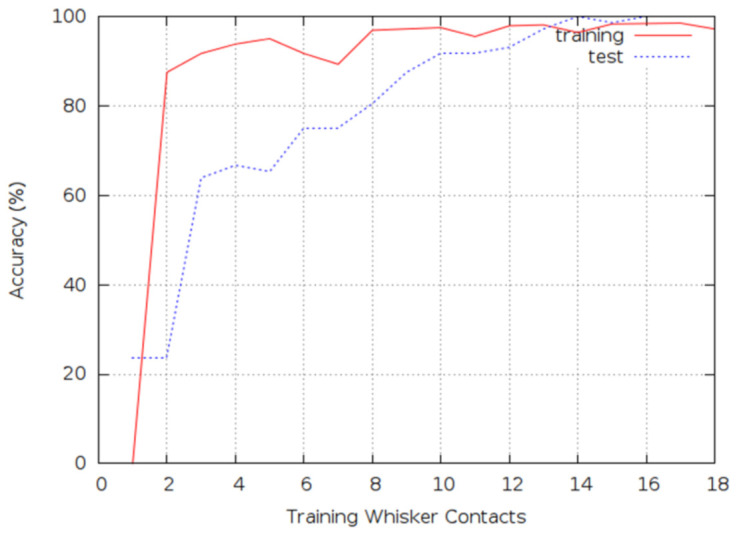
Classification accuracy for the training and test data.

In order to reduce the possibility that other surface irregularities were responsible for correct classification in recognizing the four classes of impact damage from the available four test pieces, the composite samples were rotated 90 degrees in-plane compared with the orientation during training. The 90 degrees rotation was done mainly to make sure texture of the surface ply, due to the directional properties of the composite, does not affect the classification results. It was found that progressively increasing the number of training contacts resulted in increasing classification accuracy of both cross-validation and test data. It is particularly striking that test results after training for 13 whisker contacts or more approached 100% classification accuracy as illustrated in Figure 11.

### 3.4. Discussions

From the results in Figure 11, the whisker technology was able to distinguish different impact energy levels in the specimens with an accuracy level of almost 100%. Similarly, the whisker technology was able to classify the damaged and undamaged surfaces with 100% accuracy. Even though the damage was barely visible, moving a fingertip on the impacted location, one can say if the sample was damaged or not, however, it was not possible to differentiate the impact energy levels by simply rubbing fingers on the damage location. This reflects the high capability of the whisker outperforming the manual tactile inspection both regarding the accuracy of the method and the potential for automation. It is worth noting that only the impacted face of the specimens was scanned during each experiment, which shows that simply scanning one part of the specimen could provide enough information for successful classification showing the power that the biomimetic whiskers have in terms of providing information. The whisker technology can be extended to large scale composite structures, as it detects any changes on the surface. So, for any given laminate size, impact event can be detected if it results in dents and surface texture variation. However, there will be a need for calibration and reliable training dataset for each case study.

### 3.5. Future Works

**Figure 12:**
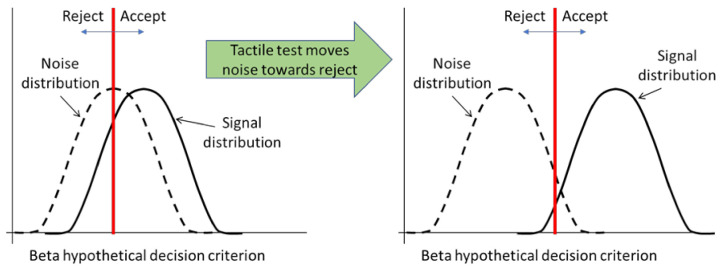
Tactile test improves visual inspection’s reliability by strengthening the “signal” component (damage) and filtering the “noise” component.

Current practice in BVID inspection is a combination of visual inspection and measuring the dent depth left by the impact. It has been reported that BVID may cause a permanent indentation of less than 0.5 mm, which is only detectable during a detailed visual inspection with a probability greater than 90% [38]. It was shown [39] that through a combination of tactile tests (running with hands over the panel surface) and continuous visual inspection it is possible to improve the inspection reliability and to find even very small damage on composite structures surface, see Figure 12. A hybrid approach can therefore be developed, by combining the whisker technology developed in this paper and visual inspection technique, to improve the inspection reliability of composite panels. Advances in automation, such as artificial intelligence technologies and inexpensive robots, have enabled the potential for automating this type of hybrid inspection system, which has the potential to surpass previous human or machine vision activities. For example, Automated unmanned aerial vehicle (UAV) based inspections could cost as little as 20% of the cost of manual visual inspections [40]. Advanced machine learning algorithms can also be developed for decision making purposes [41]. This hybrid inspection approach looks very promising for large scale composite structures such as aircraft panels and wind turbine blades, where a quick and fast visual scanning can highlight critical areas, and the whisker scanning can provide more localized and detailed information from the critical areas. This hybrid inspection approach can potentially provide accurate and reliable information on BVID and its extent, and may result in a fast, but a comprehensive scan of composite surfaces with reliable data for autonomous decision making.

## 4. Conclusions

This study investigated the feasibility of using whiskers for damage detection in laminated composite materials. These experiments have demonstrated the potential for whiskers to detect and to accurately classify BVID in composite plates according to the level of subjected impact energy. Although this procedure requires contact between the whisker tip and the test sample, that contact is brief and of very low energy. The experiments have also shown a relationship between classification accuracy and the number of whisker contacts with accuracy approaching 100% when the number of contacts exceeds 13. This suggests that the method could be applied in the detection and localization of impact damage on large composite structures provided that suitable means of deploying an array of whiskers, possibly using UAVs, could be engineered.

## Figures and Tables

**Table 1:** Characteristics of the utilized prepreg.

Prepreg Type	Cured Nominal Thickness	Ply Young Modulus E11	Fibre Failure Strain
T800 carbon/MTM49-3 epoxy	0.145 (mm)	235 (GPa)	1.70 (%)

## Data Availability

The data presented in this study are available upon request from the corresponding author.
